# Does probiotic supplementation aid weight loss? A randomized, single-blind, placebo-controlled study with *Bifidobacterium lactis* BS01 and *Lactobacillus acidophilus* LA02 supplementation

**DOI:** 10.1007/s40519-020-00983-8

**Published:** 2020-08-14

**Authors:** Dominik Czajeczny, Karolina Kabzińska, Rafał Wojciech Wójciak

**Affiliations:** 1 Chair and Department of Clinical Psychology, Poznan University of Medical Sciences, Poznan, Poland; 2 Department of Dietetics, Faculty of Physical Culture in Gorzow Wielkopolski, Poznan University of Physical Education, Poznan, Poland

**Keywords:** Microbiota, Probiotics, Weight loss, Anthropometrics

## Abstract

**Purpose:**

Probiotic supplements are gaining popularity worldwide. This trend is especially present in females, and a common motivation for consumption is weight loss, no matter the BMI. The aim of this study was to investigate the effects of probiotic supplementation on weight loss in healthy, young adult females and to put claims made by manufacturers of such products to the test.

**Methods:**

The study utilizes a randomized, single-blind, placebo-control design. 53 females aged 19–33 were enrolled, and 38 completed the trial. A 6 week supplementation with *Bifidobacterium lactis* BS01 and *Lactobacillus acidophilus* LA02 or placebo was conducted. Anthropometric measures (body mass, BMI, body fat percentage, arm skinfold fat, waist circumference, and WHR) were applied pre and post-treatment.

**Results:**

No significant changes in anthropometric measures were observed in both supplementation and placebo groups.

**Conclusion:**

The results of this investigation do not support claims made by probiotic products manufacturers, that they aid weight loss. Our results seem to support an argument that weight loss is mostly associated with food habits and dietary behaviors, not probiotic intake. It is possible that probiotic supplementation may play a facilitating weight loss but has no effect without dietary intervention. Another possible explanation is that due to strain specificity—bacteria strains used in this study are not effective for weight loss.

**Level of evidence:**

I: randomized controlled trial.

## Introduction

A growing body of research shows links between probiotic bacteria residing in the gut and the functioning of the human organism. Gut microbiota plays a crucial role in human metabolism, allowing for digesting some of the polysaccharides, amino acids, and xenobiotics, allowing for the biosynthesis of vitamins and isoprenoids [1], and for modifying mineral absorption from food [2, 3]. Changes in gut microbiota are linked to obesity and metabolical diseases [4–7]. Microbiota also plays a major role in the development of insulin resistance and type 2 diabetes by triggering low-grade inflammation [8]. There is strong evidence indicating that consumption of diets rich in saturated fat leads to increased bacterial production of pro-inflammatory lipopolysaccharide as well as gut permeability, which triggers systemic inflammation [6, 7, 9]. Data show that microbiota might be an environmental factor that plays a role in regulating fat storage. Proposed mechanisms include the ability to increase energy extraction from diet and the ability to modulate host signaling pathways (thus influencing host energy balance and metabolism) [8, 10]. But probiotics have a wide range of effects on the human organism. Reported data show positive effects of probiotics on mood [11, 12], regulation of HPA axis activity [13–15], and cognitive functioning [16, 17]. The microbiota plays an important role in the development of the immune system and can trigger an immune response [18, 19]. The mechanisms of these influences are not clearly established, and research paradigms are constantly developing. The history of research on gut microbiota was discussed broader in our previous article [9]. Data suggest that obese people have a lower diversity of intestinal bacteria compared to lean individuals and that probiotic supplementation might enrich gut microbiota composition, decrease gut permeability, inflammation, and serve as a protective factor from metabolic disorders, creating an environment which promotes weight loss [20].

There is, however, much debate around the effectiveness of probiotics. Even though probiotics are widely used with antibiotics to prevent antibiotics-associated dysbiosis, a 2018 Study by Suez et al. [21] suggests that probiotic supplementation might be actually inducing delayed and persistently incomplete microbiome reconstitution, compared to spontaneous recovery and autologous fecal microbiome transplantation (which induced near-complete recovery within days). In a study by Zmora et al. [22], luminal, mucosal, and fecal microbiome was assessed before and after administering 11-strain probiotic treatment. Results showed that the bacteria readily passed through the gastrointestinal tract into the stool and indicated a marked, person-specific mucosal colonization resistance. Zmora et al. [22] argued that this might explain high variability in probiotics efficacy.

Probiotics have become increasingly popular pharmacy and grocery items, in the form of supplements and functional foods, such as probiotic yogurt and other fermented products containing probiotics [23]. In the first half of 2015, Polish consumers bought 94.5 million packages of food supplement products, worth over 1.5 billion PLN (around 380 million USD). This was over 10 million packages more, compared to the same period of 2014, and 22 million more compared to the first half of 2012 [24]. Since 2007 (when the first food supplement was registered), around 29,000 food supplements were registered in total. Food supplements in Poland are heavily advertised. According to Prędka [25], between 1997 and 2015, the number of ads for medical products (including food supplements) and drugs increased 20-fold, and the percentage of such ads grew from 4.6 in 1997 to 24.7 in 2015. Probiotic products are the fastest-growing group of dietary supplements worldwide [26].

Despite being advertised as beneficial for health, including weight loss [27], in most countries, supplement products are considered a food [28, 29]**,** and therefore, producers are not required to prove claimed effects of the product, or even their safety [28]. Globally, the market entry requirements tend to fall under one of three categories: registration, notification, or no entry requirements [29].

Because of the rapidly increasing popularity of probiotic products in Poland [30], probiotic food supplements were chosen for this study. According to Reguła et al. [31], 43% of polish women declare using supplement products (compared to 11% of men), and weight loss is a common motivation for them, no matter the BMI [32]. The question posed in the presented investigation is whether prophylactic intake of probiotic bacteria is beneficial for changing anthropometric status in healthy, young females, especially for their weight loss?

## Methods

### Participants

53 participants were enrolled for the study via internet ads and posters on the university campus. Participants were all females, aged 19–33 (23.9 ± 3.99). During the initial interviews, basic health information was collected. Volunteers with (1) gastroenteric, (2) endocrine, (3) neurological, or (4) psychiatric disorders, and those who underwent (5) antibiotic treatment up to 3 months prior to the supplementation were excluded from the study. Volunteers who were (6) currently taking probiotic supplements were also excluded. 38 participants (20 in supplementation and 18 in the placebo group) completed supplementation and showed up for the second assessment. Reasons for leaving the study were: (1) sickness and antibiotic treatment during the period of supplementation, (2) withdrawal of consent mid-supplementation, and in one case, (3) onset of psychiatric disorder.

### Study protocol

**Fig. 1:**
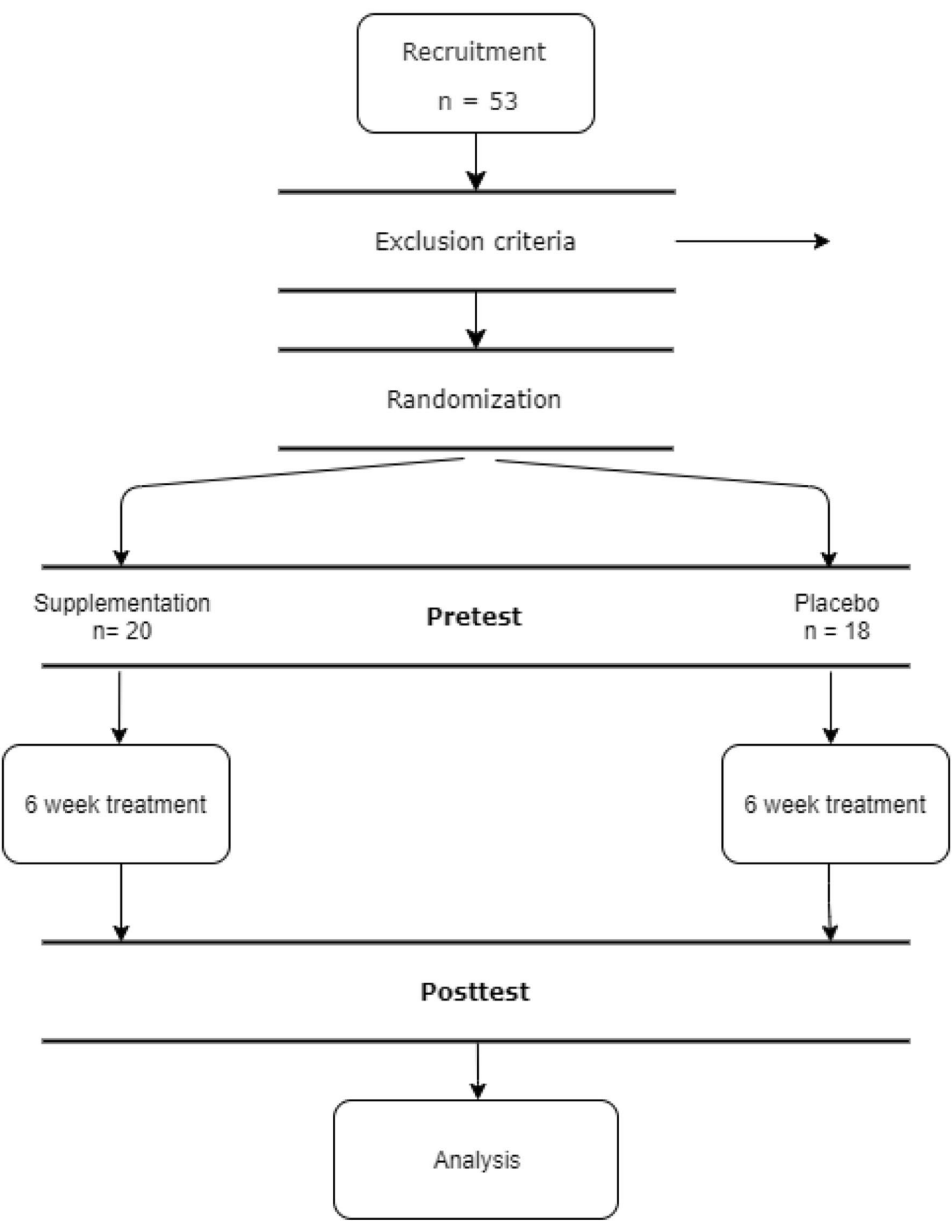
Participant flow through the study

The study utilizes a randomized, single-blind, placebo-control design. The study protocol was approved by the Poznan University of Medical Sciences Bioethics Committee (decision number 1070/16, 05.01.2017). The participant’s flow through the study is presented in Fig. 1.

Consumption of probiotic bacteria was controlled by supplementation with one of the commercially available food supplements containing *Bifidobacterium lactis* BS01 (2 × 10^9^ CFU) and *Lactobacillus acidophilus* *LA02* (2 × 10^9^ CFU) bacteria. Capsules were repacked into ziplock bags, as to remove any branding. Placebo was prepared using empty capsules similar in size and color with supplement capsules. Both products had the same taste, color, and smell. Placebo capsules were filled with maltodextrin, commonly used as a placebo in similar studies and as a carrier in probiotic supplements [11]. Randomization was performed by an experimenter using an online random number generator, selecting a random number between 1 and 2 for each participant. Participants were assigned to either supplementation or placebo group and were blinded to the assignment (single-blind design). Data were collected between October 2018 and July 2019. All measurements were conducted twice—at baseline and after treatment, by the same researcher, to minimize discrepancies between measuring techniques. Participants were instructed to take one capsule daily for 6 weeks, as recommended by the manufacturer of the supplement. The second assessment was scheduled at the convenience of the participants, but no later than 7 days after taking the last capsule. Participants were asked not to change any of their nutritional and lifestyle habits during the study. The possible changes were assessed at the end of the study using 24 h recall. There were no significant differences in energy and nutrients intake, as well as the structure of product consumption by participants. Participants were instructed to immediately contact the researcher in case any side effects occur. No participants reported any side effects during or after the study; only one participant complained about capsules being difficult to swallow due to their size.

### Sample size calculations

To calculate a priori sample size, a standard formula suggested for parallel clinical trials [33] with type one error (*α*) of 0.05 and type two (*β*) of 0.20 (power 80%) was used. Based on a previous study [33], a mean difference in BMI (primary outcome of this study) of 0.2 and SD of 0.2 was applied. Based on this, 11 subjects in each group were needed. We decided to aim for double the necessary size, considering other outcomes included in the study and the expected drop-out rate.

### Anthropometric measures

Participants were asked to stand on a scale without shoes and in light clothing. Body mass was assessed using medical, electronic stale with 0.1 kg accuracy. Body height was measured using a measuring rod to the nearest 1 cm. Body mass index was calculated as weight (kg) divided by the square of height (m). Participants were classified according to BMI as underweight (BMI < 18.5 kg/m^2^) normal weight (BMI < 25.0 kg/m^2^), excess body weight (BMI > 25 kg/m^2^) [16].

Waist circumference was measured midway between the lowest rib and iliac crest using a non-stretchable measuring tape. Hips circumference was measured as the widest part of the hips, around the widest portion of the buttocks. Waist to hip ratio (WHR) was calculated by dividing waist measurement in cm by the hips measurement in cm. Participants were classified according to waist circumference as not at risk of metabolic complications (waist circumference < 80 cm), at increased risk of metabolic complications (waist circumference 80–88 cm), and at substantially increased risk of metabolic complications (waist circumference > 80 cm). Participants were also classified according to WHR as not at substantially increased risk of metabolic complications (WHR < 0.85) or at substantially increased risk of metabolic complications (WHR > 0.85) [34]

Body composition (percentage of fat tissue) was assessed using a handheld bioimpedance device (Clatronic FAG 2694) and referred to values provided by the manufacturer.

Arm skinfold fat was measured at triceps using standard calipers [34].

### Statistical analyses

Due to the small sample size, a non-parametric Wilcoxon signed rank test was used to calculate the significance of changes pre and post supplementation. Effect sizes for the Wilcoxon signed rank test were calculated using $r = \frac{Z}{\sqrt N }$. Wherever reference values were applicable, additional Chi-square tests were conducted to test whether the sample characteristics differed significantly pre and post-treatment. Effect sizes for Chi-square tests were calculated using Cramer’s V.

## Results

Table 1 shows changes in anthropometric measures pre and post-treatment. Body mass decreased more after treatment in the supplementation group (by 3.34%, compared to 0.60% in the placebo group), but the differences were not significant. Similarly, BMI decreased more after treatment in the supplementation group (by 4.1%, compared to 0.81% in the placebo group), but the differences were not significant. Body fat percentage increased in the supplementation group by 0.70%, but the change was not statistically significant. In the placebo group, body fat percentage decreased by 1.95%, but this change was also not statistically significant. Arm skinfold measured at the triceps decreased more in the placebo group (by 10.87%, compared to 1.45% in the supplementation group). Both changes were not statistically significant. Waist circumference increased in the supplementation group by 0.67% and decreased in the placebo group by 1.33%. In both groups, changes were not statistically significant. Similarly, WHR increased in the supplementation group by 1.195% and decreased in the placebo group by 1.36%. Changes were also not significant. All the effect sizes were small.

**Table 1:** Changes in anthropometric measures pre and post-treatment

	Supplementation					Placebo				
	Pre	Post	Change %	*P* value	*r*	Pre	Post	Change %	*P* value	*r*
Body mass (kg)	67.4 ± 16.1	65.2 ± 12.9	− 3.3	0.936	0.13	61.1 ± 7.6	60.8 ± 8.9	− 0.6	0.164	0.25
BMI	24.2 ± 5.8	23.2 ± 3.5	− 4.1	0.872	0.14	22.0 ± 2.4	21.8 ± 3.0	− 0.8	0.125	0.27
Body fat (%)	29.6 ± 5.6	29.7 ± 5.5	0.2	0.695	0.06	27.9 ± 3.982	27.4 ± 4.6	− 2.0	0.083	0.32
Arm skinfold fat (mm)	24.2 ± 7.7	23.9 ± 6.6	− 1.4	0.642	0.07	24.9 ± 7.1	22.2 ± 6.8	− 10.9	0.220	0.22
Waist circumference (mm)	74.9 ± 9.1	75.3 ± 8.6	0.7	0.374	0.14	72.1 ± 5.4	71.2 ± 6.0	− 1.3	0.765	0.05
WHR	0.753 ± 0.055	0.762 ± 0.053	1.2	0.232	0.28	0.738 ± 0.030	0.728 ± 0.038	− 1.4	0.281	0.19

Data are mean ± SD

*BMI* body mass index, *BF* body fat, *WHR* waist to hip ratio

Wherever reference values were applicable, additional Chi-square tests were conducted to test whether the sample characteristics differed significantly pre and post-treatment. The results are shown in Table 2. Overall, changes in sample characteristics pre and post-treatment were not statistically significant on all measures, with small effects sizes.

**Table 2:** Percentage distribution of anthropometric parameters values between participants pre and post treatment

Parameter	Supplementation		Chi-square test result	Cramer’s V	Placebo		Chi-square test result	Cramer’s V
	Pre	Post			Pre	Post		
BMI [kg/m^2^]			NS	–			NS	–
< 18.49	0	0			5	5		
18.5–24.99	70	70			78	78		
> 25	30	30			17	17		
WHR			NS	0.09			NS	0.16
< 0.85	90	95			100	95		
> 0.85	10	5			0	5		
Body fat [%]							NS	0.06
< 17	0	0	NS	0.07	0	0		
17–24	25	25			22	22		
24.1–30	35	40			50	45		
30.1–40	40	35			28	33		
> 40	0	0			0	0		
Waist circumference [cm]			NS	0.15			NS	0.08
< 80	75	70			83	78		
80–88	15	25			11	16		
> 80	10	5			6	6		

*NS* not significant

In the supplementation group at pretest, no participants were classified as underweight, 70% were classified as normal weight, and 30% as overweight. No changes were observed in percentage distribution after treatment. In the placebo group at pretest, 5% of participants were classified as underweight, 78% were classified as normal weight, and 18% as overweight. No changes were observed in percentage distribution after treatment.

In the supplementation group at pretest, 90% of participants were classified as not at risk of metabolic diseases (WHR < 0.85) and 10% as at risk (WHR > 0.85). After treatment, percentage distribution shifted towards not at risk category (95% < 0.85; 10% > 0.85). In the placebo group at pretest, 100% of participants were classified as not at risk of metabolic diseases (WHR < 0.85) and 0% as at risk (WHR > 0.85). After treatment, 5% of participants were classified at-risk category (95% WHR < 0.85; 10% WHR > 0.85). These changes were not statistically significant.

In the supplementation group at pretest, no participants were classified as below recommended BF content (< 17), 25% as ideal (17–24), 35% as recommended (24.1–30), 40% as excessive (30.1–40) and no participants were classified as highly excessive (> 40). After treatment, a shift towards recommended BF content was observed (40% classified as normal; 35% as excessive). In placebo group at pretest, no participants were classified as below recommended BF content (< 17), 22% as ideal (17–24), 50% as recommended (24.1–30), 28% as excessive (30.1–40) and no participants were classified as highly excessive (> 40). After treatment, a shift towards excessive was observed (45% classified as recommended; 33% as excessive). These changes were not statistically significant.

In the supplementation group at pretest, 70% of participants were classified as normal central fat accumulation (< 80 cm), 15% was classified as moderate central fat accumulation (80–88 cm), and 10% as high central fat accumulation (above 88 cm). A shift towards moderate central fat distribution was observed after treatment (70% < 80 cm; 25% 80–88 cm; 5% > 88 cm). In the placebo group at pretest, 83% of participants were classified as normal central fat accumulation (< 80 cm), 11% was classified as moderate central fat accumulation (80–88 cm), and 6% as high central fat accumulation (above 88 cm). A shift towards moderate central fat distribution was observed after treatment (78% < 80 cm; 16% 80–88 cm; 6% > 88 cm). These changes were not statistically significant.

## Discussion

Recently, an increase in the consumption of food supplements is observed, especially in females. This trend is present in all age groups, and a common motivation for using such products is aiding weight loss—including body mass, body fat content, and body size decrease. Probiotic supplements are among the most popular products advertised and used for this purpose.

The presented study investigated the effects of prophylactic *B. lactis* BS01 and *L. acidophilus* LA02 supplementation on anthropometric measures in healthy, young females. The main aim of the study was to assess whether prophylactic consumption of probiotic supplements can aid weight loss. Results show no significant effects of both probiotic treatment and placebo on all anthropometric measures (Table 1): body mass, BMI, body fat percentage, arm skinfold fat, waist circumference, and WHR. Wherever reference values were applicable, participants were scored accordingly (Table 2) to test whether the percentage distribution of the study group was affected by the treatment. Also, in these analyses (BMI, WHR, BF, waist circumference), no significant changes were observed. These findings are in line with most published data [2, 14, 19, 25, 29, 30]. All the effect sizes obtained in the study were small, further supporting this outcome.

Few studies emphasized the role of gut microbes in obesity and their crucial role in the development of obesity [5, 41, 42]. Despite this, Park and Bae [27] argued that weight loss is mostly associated with food habits and dietary behaviors, not probiotic intake.

In a study by Zarrati et al. [35], obese participants were on a low-calorie diet and were receiving either probiotic (containing *L. acidophilus* La5, *Bifidobacterium* BB12, and *Lactobacillus casei* DN001) or regular yogurt (control group received only probiotic yogurt with no diet modification). Probiotic treatment without a low-calorie diet had no effect on anthropometric measures. Both dieting groups showed a decrease in some of the anthropometric measures, after 8 weeks of treatment. The study reported a greater decrease in body weight, BMI, weight, and hip circumference in a group that received diet and probiotic yogurt compared to the diet-only group, but these differences were not statistically significant. The study failed to find changes pre and post-treatment in WHR and Mid‐Upper Arm Circumference in both dieting groups.

Sanchez et al. [43] reported positive effects of synbiotic supplementation (a combination of *Lactobacillus rhamnosus* CGMCC1.3724 and prebiotics—oligofructose and inulin) on weight loss. Weight loss was observed after 12 weeks of synbiotic supplementation and moderate energy restriction, and further weight loss was observed in the following 12 weeks of the weight maintenance period. In females who received a placebo, weight and fat mass after 12 weeks were significantly lower, but weight and fat mass gain were observed after the following 12 weeks of the weight maintenance period. Changes were observed only in female participants. The study outcome suggests that the effects of probiotics might be sex-specific, but to our knowledge, no other data supporting this were published yet. In both studies above, participants were obese at baseline, and beside probiotic supplementation, a dietary intervention was conducted. Results point towards a facilitating role of probiotics in weight loss and maintenance.

A study by Sergeev et al. [44] provides evidence that high-protein and low-carbohydrate diets, which are often successfully used for weight loss, have been associated with a decrease in bacteria considered beneficial to health, and there is a possibility that probiotic supplementation helps to restore gut microbiota, contributing to weight loss [44]. Results reported by Sergeev et al. [44], although promising, were obtained with the use of synbiotic, not probiotic supplementation (a combination of *L. acidophilus* DDS-1, *B. lactis* UABla-12, *Bifidobacterium longum* UABl-14, *Bifidobacterium bifidum* UABb-10 and a prebiotic component—a *trans*-galactooligosaccharide mixture).

A large study showing positive effects of probiotic treatment on anthropometric measures was published by Kadooka et al. [36]. The study reported a significant decrease in abdominal visceral fat areas (which were determined by computed tomography) BMI, waist and circumference, and body mass after 12 weeks of probiotic supplementation with *Lactobacillus gasseri* SBT2055. No significant changes were found in the placebo group. Cessation of probiotic treatment attenuated these effects after 4 weeks, suggesting that constant consumption might be needed to maintain the effect. Positive effects of probiotic supplementation were also found in a study by Ahmadi et al. [33]. After 12 weeks of supplementation with a combination of *L. acidophilus* (2 × 10^9^ CFU/g), *Lactobacillus casei* (2 × 10^9^ CFU/g) and *B. bifidum* (2 × 10^9^ CFU/g) (authors provided doses, but not specific strain designations of bacteria used in the study), with no diet modification, a decrease in body mass and BMI was observed in 30 females with polycystic ovary syndrome. No changes were observed in the placebo group.

A meta-analysis of 25 randomized, placebo-controlled trials by Zhang et al. [45] concluded that probiotic consumption significantly reduced body weight, with greater reduction resulting from multi-species probiotics and treatment period over 8 weeks, in participants overweight and obese at baseline. A recent meta-analysis of 43 trials by Koutnikova et al. [46] also found small but significant effects of probiotics on weight loss. However, improvement in some anthropometric (body fat mass and waist circumference) measures was observed in overweight, but not obese patients, suggesting that due to severe gut microbiota dysbiosis associated with obesity, these patients might be resistant to probiotic supplementation, or might require long-term treatment.

A review of clinical trials by Marques et al. [20] identified only two studies (from 13 clinical trials selected), both using *L. gasseri* (strains SBT2055 and BNR17) in doses between 1 × 10^6^ and 1 × 10^10^ CFU, which reported body fat reduction. Authors point towards two main problems with studies on the effects of probiotics: strain-specific effects of probiotics and the fact that most of the studies also include either dietary intervention (hypocaloric diet) or do not control caloric intake sufficiently, along with probiotic supplementation, making it difficult to assess the effects of probiotics alone.

A meta-analysis by McFarland et al. [47] found strong evidence that the efficacy of probiotics is both strain-specific and disease-specific. Strain-specific efficacy for preventing adult antibiotic-associated diarrhea (AAD) was found within the *Lactobacillus* species, but not all *Lactobacillus* species showed efficacy for preventing AAD. Even directly compared, two different strains of the same species (*Lactobacillus casei*) showed a significant difference in efficacy for preventing AAD. It is possible then that results obtained in this study were not significant due to chosen strains not being effective for influencing host energy balance and metabolism (and thus weight loss). According to McFarland et al. [47], some of the *L. acidophilus* (one of the strains used in this study) are successfully used for preventing AAD and *Clostridium difficile* infections. This means that a combination of *B. lactis* BS01 and *L. acidophilus* LA02 used in this study might be not effective for weight loss, but still beneficial for other health parameters.

As we described broader in our previous article [9], microbial components responsible for health benefits are still unknown [48], and the effects of probiotic products are difficult to study because of person-specific resistance to mucosal colonization in the gut after administering probiotic bacteria described by Zmora et al. [22], a multitude of bacteria strains (and combinations of strains with/without prebiotics) used for research and difficulties insufficient diet control during the trial. Not all bacteria strains might produce similar outcomes. These might be important confounding factors in research on the effects of probiotics on human health. Some studies indicate that rather than probiotic supplementation, complete fecal microbiome transplantation (autologous or from a healthy donor) might be a more reliable way of modulating gut microbiota composition [21, 49]. A meta-analysis by Kristensen et al. [50] states that there is no evidence that probiotic supplementation alters fecal microbiota composition compared to placebo. Simple probiotic supplementation, while not harmful, might not be effective in altering gut microbiota, or at least not universally [21].

None of the studies mentioned above reported any adverse effect of probiotic products [33, 35–40, 43], and none were found in this study. Food supplements and functional food are deemed generally safe.

The outcome of this study does not support the claims by the producers of probiotic supplements that daily, prophylactic consumption of such products is beneficial for weight loss. However, the probiotic supplement used in this study might be effective in other areas (e.g., for AAD prevention). De Simone [51] argues that manufacturers take advantage of the “probiotic umbrella”, by extending the results obtained with a specific product, dose, duration of intake, a combination of strains, methods used to manufacture the formulation and research group to all probiotic products. Because of the unregulated nature of food supplements in general, this transfer is not warranted and may be confusing to the customers.

Strengths of the study include placebo-controlled, single-blind design, narrowing the sample to participants aged 20–30, a relatively long time of supplementation, and control over caloric intake. Participants with possible disease-related (and after antibiotic treatment) alterations in gut microbiota were excluded from the study, ensuring similar baseline conditions for supplementation. The second assessment was conducted shortly after the end of the supplementation period, as data suggests that discontinuing treatment might reverse its effects [20].

A possible weakness of this study is a relatively small sample and no control over metabolic and inflammation parameters. According to Marques et al. [20], in order to further our understanding of probiotic supplementation effects on weight loss, very precise control over caloric intake, type of nutrients consumed, level of insulin sensitivity and inflammation parameters needs to be ensured.

## What is already known on this subject?

Gut microbiota is linked to the development of obesity and metabolical disorders, but the mechanisms behind this association have not been clearly established. Probiotic products are gaining popularity, mostly because of the broadly advertised benefits for weight loss. There is some evidence that probiotic supplementation might be beneficial for weight loss, but the results are not conclusive, and published trials are limited by insufficient control over diet and other confounding factors. Most of the studies introduce a dietary intervention along with probiotic treatment, making it difficult to assess the effects of probiotics alone.

## What does this study add?

The study provides evidence that prophylactic probiotic supplementation without a hypocaloric diet does not cause weight loss in healthy young adults, further supporting the hypothesis that probiotics might play a facilitating role for weight loss. The study points towards possible confounding factors necessary to control in further research. Probiotic supplementation with commercially available products while not harmful, might not be beneficial for health, including weight loss.
